# The effect of *Channa striata* extract and standard eradication regimen on asymmetric dimethylarginine in *Helicobacter pylori* gastritis rat model

**DOI:** 10.14202/vetworld.2020.1605-1612

**Published:** 2020-08-17

**Authors:** OK Yulizal, Aznan Lelo, Syafruddin Ilyas, Raden Lia Kusumawati

**Affiliations:** 1 Department of Internal Medicine, Faculty of Medicine, Universitas Prima Indonesia, Medan, Indonesia; 2 School of Medicine, Universitas Sumatera Utara, Medan, Indonesia; 3 Department of Pharmacology and Therapeutics, School of Medicine, Universitas Sumatera Utara, Medan, Indonesia; 4 Department of Biology, Faculty of Mathematics and Natural Sciences, Universitas Sumatera Utara, Medan, Indonesia; 5 Department of Microbiology, School of Medicine, Universitas Sumatera Utara, H. Adam Malik General Hospital, Medan, Indonesia

**Keywords:** asymmetric dimethylarginine, *Channa striata*, eradication regimen, *Helicobacter pylori* gastritis, histological changes

## Abstract

**Background and Aim:**

The presence of gastric mucosa or submucosa inflammation due to *Helicobacter pylori* leads to histological changes. Gastric injury, pro-inflammatory factors, and oxidative stress in *H. pylori* infection produce asymmetric dimethylarginine (ADMA), which are a competitive inhibitor of nitric oxide synthase. Investigations were carried out aimed at finding new drugs derived from natural products for the treatment of *H. pylori*. *Channa striata* is known to have *in vitro* anti-inflammatory and antimicrobial properties. This study aimed to investigate the effect of *C. striata* extract and a standard eradication regimen on ADMA levels and histological changes in the *H. pylori* gastritis rat model.

**Materials and Methods:**

Thirty-five male rats were randomly and equally divided into five groups. Group-1 was the negative control group and Groups-2 to 5 were *H. pylori*-infected groups. Groups-3 to 5 were administered *C. striata* extract, a standard eradication regimen, and a combination of standard eradication regimen and *C. striata* extract, respectively. Histological examination and serum ADMA levels were analyzed. The difference between groups was analyzed using the Kruskal–Wallis and one-way analysis of variance tests. The significance was p<0.05.

**Results:**

Serum ADMA levels and severity of gastritis were higher in infected groups compared to the negative control group (p<0.05). The severity of gastritis and mean ADMA levels in the group that received a single administration of the *C. striata* extract was higher than the others (p<0.05). Serum ADMA levels and severity of gastritis were significantly reduced in the group that received a combination of standard eradication regimen and *C. striata* extract (p<0.05).

**Conclusion:**

Single administration of *C. striata* extract worsens the severity of gastritis and increased serum ADMA levels in the *H. pylori* gastritis rat model. The administration of a combination of standard eradication regimen and *C. striata* extract reduces serum ADMA levels and significantly improves the severity of *H. pylori* gastritis rat model.

## Introduction

*Helicobacter pylori* is a bacterium that causes damage to the gastric mucosa, commonly in the form of gastritis, peptic ulcer disease, or even gastric malignancy [1]. Gastritis is the most common clinical manifestation of *H. pylori* infection. The occurrence of gastric mucosa or submucosa inflammation due to *H. pylori* causes mild-to-severe histological changes. The degree of gastritis is related to the density of *H. pylori*, gastric mucosal injury, and inflammation [2]. Gastric mucosal injury is characterized by an increase in endogenous nitric oxide synthase (NOS) inhibitor levels, namely, asymmetric dimethylarginine (ADMA) [3]. An increase in ADMA levels leads to the reduction of nitric oxide (NO) through the inhibition of NOS activity, and an enzyme generates NO from arginine [4]. NO plays a role in preserving the gastric mucosa through the maintenance of mucosal perfusion, and some studies have noted that ADMA could be a marker for gastric mucosal injury. ADMA is a substance that plays a role in the process of atherogenesis and is closely associated with diseases linked to that process [5]. Inflammation is characterized by changes in cytokines, especially pro-inflammatory cytokines. Changes in these cytokines will ultimately bring about increased ADMA production [6].

Management of *H. pylori* infections has progressed since this bacterium was popularized by Barry Marshall and Robin Warren several decades ago. The discovery of several *H. pylori* eradication treatment regimens continues to be the aim of finding new drugs as well as increasing the effectiveness of existing eradication regimens. There are several known reasons for the reduced efficacy of the *H. pylori* standard eradication regimen, including patient compliance, immunity status, nutrition, and antimicrobial resistance [7]. The reduced effectiveness of the *H. pylori* standard eradication regimen causes antimicrobial resistance, a severe problem mainly in areas with high levels of antimicrobial resistance [8]. In an effort to eradicate *H. pylori* and to reduce the level of antimicrobial resistance, investigations are currently being conducted to find new drugs that come from natural sources, including plant and animal extracts, for use as a single drug or in combination with current eradication regimens. The selection of alternative and additional drugs for the treatment of *H. pylori* infection is also aimed at reducing the side effects of certain medications, lowering treatment costs, and assessing the antimicrobial and anti-inflammatory properties of the extracts [7]. *Channa striata* is a species of predatory fish that lives in the Asia Pacific region and is widely consumed by its inhabitants [9]. *C. striata* extract is a source of animal protein and is believed to contain nutrients that are important in improving body stamina after labor, surgery, and healing after suffering from certain diseases. Researchers have characterized the high nutrient content of this fish, including the protein, albumin, amino acid, fatty acid, vitamin, and mineral content. All of these nutrients are very useful in the prevention and treatment of several diseases [10]. *C. striata* extract reportedly has anti-inflammatory, antioxidant, and anti-gastric ulcer properties [9,11]. Several studies have documented the potential role of *C. striata* extract *in vitro* as an antimicrobial and antifungal [12,13].

Based on the *in vitro* antimicrobial properties and *in vivo* anti-inflammatory properties of *C. striata* extract, we are interested in testing its potential for reducing injury to the gastric mucosa by assessing the ADMA levels and the histological features in the *H. pylori* gastritis rat model. This study aimed to investigate ADMA levels and histological changes after administration of *C. striata* extract as a single drug and or in combination with the *H. pylori* standard eradication regimen in the *H. pylori* gastritis rat model.

## Materials and Methods

### Ethical approval

All experiments and procedures of this research were conducted after gaining approval from the Animal Research Ethics Committee, Department of Biology, Faculty of Mathematics, and Natural Sciences, Universitas Sumatera Utara, Medan, Indonesia, with approval number 0448/KEPH-FMIPA/2019.

### Study period and location

This study was performed from October to December 2019 at Microbiology Laboratory, Biomedical Research Unit, West Nusa Tenggara Province General Hospital, Indonesia.

### Study design

This experimental study used a post-test only with the control group design and determination of samples by simple random sampling.

### Experimental animals

This study involved 35 male albino rats of the species *Rattus norvegicus* Wistar strain purchased from Biomedical Research Unit, West Nusa Tenggara Province General Hospital. The rats were 8-12 weeks old, with mean body weight (BW) of 294 g, and showing active movement and good appetite. They were housed in cages subjected to a 12 h light/dark cycle, at room temperature 27±2°C with 70-80% humidity. The cages were sanitized regularly, fed with standard commercial rodent food, and provided with water *ad ­libitum*. The rats were acclimated for 7 days in the laboratory, then divided randomly into five groups of seven each, namely: (1) Group-1 (negative control, without *H. pylori* inoculation); (2) Group-2 (positive control, *H. pylori* infection rat model); (3) Group-3 (*H. pylori* infection rat model + *C. striata* extract with a dose of 300 mg/kg BW); (4) Group-4 (*H. pylori* infection rat model + standard eradication regimen (aqueous solution of amoxicillin 50 mg/kg BW + clarithromycin 25 mg/kg BW + omeprazole 20 mg/kg BW); and (5) Group-5 (*H. pylori* infection rat model + a combination of standard eradication regimen (aqueous solution of amoxicillin 50 mg/kg BW + clarithromycin 25 mg/kg BW + omeprazole 20 mg/kg BW) and *C. striata* extract with a dose of 300 mg/kg BW.

### *H. pylori* preparation

The *H. pylori* isolate was obtained from the gastric biopsy specimen of a duodenal ulcer patient, which was kept and cultured at the Microbiology Laboratory, Biomedical Research Unit, West Nusa Tenggara Province General Hospital. *H. pylori* from this human isolate was cultured using Tryptic Soy Agar to which 10% fresh sheep blood, 2 mL/500 mL Dent supplement, and 10 mL/500 mL Vitox supplement (Oxoid™, Thermo Scientific™, Hampshire, UK) were added. Incubation was carried out in a CO_2_ incubator under microaerophilic atmospheric conditions with a concentration of 5% O_2_, 10% CO_2_, and 85% N_2_ for 72 h at 37°C. Then, the colonies suspected of being *H. pylori* were confirmed by appearance, microscopic Gram-staining, and biochemical analysis (oxidase, catalase, and urease). The rats in Groups 2, 3, 4, and 5 were inoculated with *H. pylori* suspension containing 5 × 10^8^-5 × 10^10^ colony-forming units/mL equivalent to 2.0 McFarland standard in 0.9% NaCl at 1 mL/rat.

### *C. striata* extract and standard eradication regimen

The *C. striata* extract was obtained from Channa^®^ capsules (PJ. Herbal Nusantara, Solo, Indonesia) and purchased from a retail drug store. Each 500 mg capsule contained pure *C. striata* extract powder. The biochemical compounds of Channa^®^ capsules were analyzed at the Department of Pharmacy, Universitas Muhammadiyah Surakarta, Middle Java, Indonesia. *C. striata* extract was dissolved in 0.5% (w/v) carboxymethyl cellulose (0.5% CMC) and administered orally by intragastric tube at 1 mL/rat once a day. A dose of 300 mg/kg BW was used as a treatment dose, which was converted from a human dose, as reported by an earlier study [14]. The standard eradication regimen consisted of amoxicillin, clarithromycin, and omeprazole (PT. Indofarma, Jakarta, Indonesia) in aqueous solution (dissolved in 0.5% CMC) at a dose of 50 mg/kg BW, 25 mg/kg BW, and 20 mg/kg BW, respectively, all purchased from a commercial drug store.

### Experimental procedure

After 7 days of the acclimation period, rats in Group-1 were given ordinary drinking water, while Groups-2 to -5 were pre-treated with streptomycin (PT. Indofarma, Jakarta, Indonesia) suspended in tap water (5 mg/mL) for 72 h prior inoculation of *H. pylori*. The streptomycin was purchased from a retail drug store. All rats then fasted for 24 h the next day, and Group-1 was supplemented with a solution of 0.9% (w/v) sodium chloride (0.9% NaCl) at 1 mL/rat orally by intragastric tube twice a day at an interval of 4 h for 3 consecutive days. In addition, Groups-2 to -5 were inoculated with an aqueous 1 mL/rat suspension of *H. pylori* orally by intragastric tube twice a day at an interval of 4 h for 3 consecutive days. Three hours before the first of *H. pylori* inoculation and during the following 6 days, the rats in Groups-2, 3, 4, and 5 were administered omeprazole (PT. Indofarma, Jakarta, Indonesia) orally, at a dose of 400 μmol/kg BW dissolved in 0.5% CMC, purchased from a commercial drug store, and 1 mL/rat once a day orally by intragastric tube, whereas the rats in Group-1 were given a 0.5% CMC suspension following the same procedure.

Two weeks after the last day of *H. pylori* inoculation, all rats fasted overnight before treatment. A rat from each group was randomly selected and sacrificed under diethyl ether anesthesia in a chamber for the monitoring *H. pylori* infection. A rat selected from each group was carried out to a midline laparotomy device. The stomach was removed, gastric mucosa from the antral area (2 mm^2^) was cut out, and a urease test (Pronto Dry^®^, Gastrex, France) was performed for the detection of *H. pylori* in gastric tissue. This procedure produced a positive urease test in all rats that represented the inoculation groups.

The next day, the remaining rats from each group were treated accordingly. The rats in Groups-1 and -2 were administered 0.5% CMC suspension orally once daily by intragastric tube for 7 days. Meanwhile, the rats in Group-3 received the *C. striata* extract orally 1 mL/rat once daily through an intragastric tube for 7 days. The rats in Group-4 were administered the standard eradication regimen orally at 1 mL/rat for 7 days, and the rats in Group-5 were administered the standard eradication regimen and 1 mL/rat *C. striata* extract at an interval of 6 h orally, respectively, once daily by intragastric tube for 7 days. All rats were sacrificed under diethyl ether anesthesia in a chamber, 4 weeks after this treatment protocol. The laparotomy procedure was followed to perform a rapid urease test, as in the previous description. All animals were successfully infected with *H. pylori* in the infected groups. Furthermore, gastric antral was provided for histological examination, and the blood was taken directly from the heart.

### Histological and ADMA examination

Gastric mucosal tissue in a small fragment from the antral region was removed and fixed with 10% buffered neutral (pH 7.4) formalin solution for 24 h, and tissue processing and embedding were then carried out on the paraffin block. The paraffin block was cut to a thickness of 5 μm and placed on slides before staining. The procedure was continued using the hematoxylin-eosin staining technique. Microscopic examination was conducted blindly by a pathologist. The severity of gastritis and bacterial colonization was scored and classified using the updated Sydney system (USS) to assess lymphocyte infiltration, neutrophil activity, glands atrophy, intestinal metaplasia, and density of *H. pylori* [15].

The blood from each rat was collected through a cardiac puncture and allowed to clot for 2 h at room temperature. The clotted blood was then centrifuged at 3000 revolutions/min (rpm) for 20 min to obtain serum on the ADMA examination. The serum was removed and frozen at −70°C until the day of analysis. The enzyme-linked immunosorbent assay kit (Cat. No. E0137Ra, Bioassay Technology Laboratory, Shanghai, China) was used to measure the ADMA levels accordingly.

### Statistical analysis

The Shapiro–Wilk test was used to evaluate the normality of the quantitative variables. The results were presented as mean±standard deviation for normally distributed data and median ­(minimum-maximum) for non-normally distributed data. We computed the differences between the mean of quantitative variables with normal distribution using one-way analysis of variance (ANOVA) with *post hoc* Tamhane test and Kruskal–Wallis test for non-normal data distribution to compare and analyze data. The statistical software SPSS version 22.0 (IBM, Chicago, IL, USA) was utilized to perform computation, and p<0.05 was determined as the significance of the limit.

## Results

**Figure-1:**
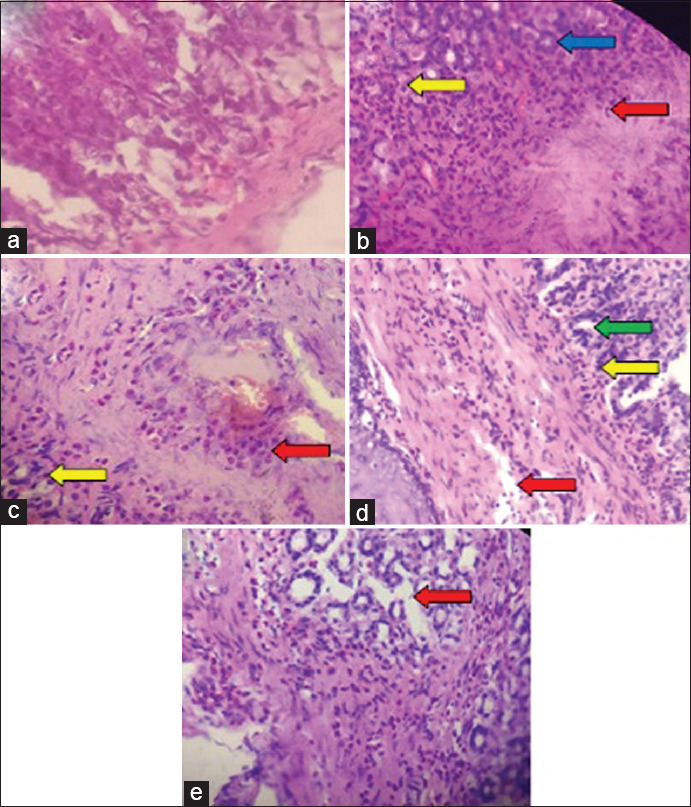
Histological representation of rat’s gastric tissue (Hematoxylin and Eosin staining). (a) Group-1; (b) Group-2; (c) Group-3; (d) Group-4; (e) Group-5. Red arrows indicated *Helicobacter pylori*; yellow arrows indicated polymorphonuclear leukocytes cells; blue arrows indicated edema; green arrows indicated vasodilation. (Olympus^®^ CX22 LED microscope with a magnification of 400× and photomicrographs were carried out with a Vivo^®^ 9 camera).

The severity of gastritis was assessed in this study through histological examination based on the USS. A tabulation of histological examination results is presented in Table-1. It shows more neutrophil cells in Groups-2 and -3 compared to the other groups (Kruskal–Wallis test, p<0.05). In contrast, intestinal metaplasia and *H. pylori* density were more common in Group-3 than in other groups (Kruskal–Wallis test, p<0.05). Histologically, the severity of gastritis was higher in Group-3, followed by Groups-2, -4, -5, and -1, respectively (Kruskal–Wallis test, p<0.05). Based on the results of the histological examination in rats of all groups, we found as many as 7% normal ones, 50% with mild gastritis, and 43% with moderate gastritis. No rat was classified as severe gastritis. In Figure-1, we present the microscopic images of gastric antral tissue in the histological examination.

**Table-1:** Tabulation of histological examination results based on the USS.

Groups	Variable					USS total score	p-value

	Lymphocyte	Neutrophil	Glands atrophy	Intestinal metaplasia	*H. pylori* density		
Group-1	0.17±0.41	1.00±0.89	0.00±0.00	0.00±0.00	0.00±0.00	1.17±1.17	<0.001*
Group-2	1.17±0.41	2.17±0.41	0.83±0.41	0.00±0.00	1.33±0.52	6.50±0.55	
Group-3	1.17±0.41	2.33±0.52	1.17±0.41	0.83±0.41	2.33±0.52	7.83±1.47	
Group-4	1.17±0.41	1.67±0.82	0.50±0.55	0.50±0.55	1.33±0.52	5.17±0.75	
Group-5	0.67±0.52	1.33±0.52	0.67±0.52	0.00±0.00	1.00±0.63	3.67±1.03	

Data expressed as mean±SD. Statistical test using one-way ANOVA. *p<0.05. USS=Updated Sydney System, SD=Standard deviation, ANOVA=Analysis of variance, *H. pylori=Helicobacter pylori*

**Figure-2:**
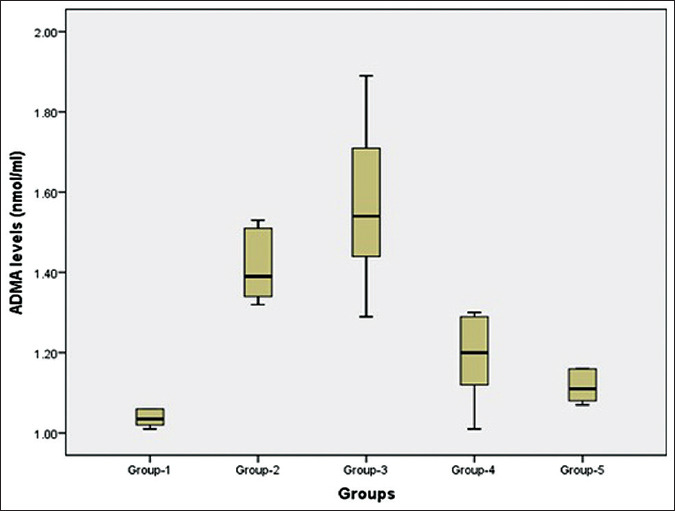
Boxplot asymmetric dimethylarginine levels of the experimental groups.

Figure-2 shows the ADMA levels of each group. It was demonstrated that the mean ADMA levels in Group-3 were the highest compared to the other groups. The details of the difference are displayed in Table-2. A statistical test using one-way ANOVA showed a difference in the mean of ADMA levels between groups (p<0.001). It demonstrated that the mean of ADMA levels in Group-3 was the highest, while the mean of ADMA levels in Group-1 was the lowest compared to the other groups. To determine the differences in the mean of ADMA levels between groups, a *post hoc* Tamhane analysis was carried out. The results of the analysis are displayed in Table-3.

**Table-2:** The mean of ADMA levels in each group.

Experimental Groups	n	ADMA (nmol/mL)	p-value
Group-1	6	1.04±0.02	<0.001*
Group-2	6	1.41±0.09	
Group-3	6	1.57±0.21	
Group-4	6	1.19±0.11	
Group-5	6	1.12±0.04	

The results were mean±SD. Statistical test using one-way ANOVA.

* p<0.05. ADMA=Asymmetric dimethylarginine, SD=Standard deviation, ANOVA=Analysis of variance

**Table-3:** *Post hoc* analysis in comparing ADMA levels in each group.

Groups	Mean difference	95% CI		p-value

		Minimum	Maximum	
Group-1 versus Group-2	−0.38	−0.55	−0.21	0.001*
Group-1 versus Group-3	−0.53	−0.93	−0.13	0.015*
Group-1 versus Group-4	−0.15	−0.36	0.06	0.179
Group-1 versus Group-5	−0.08	−0.15	−0.01	0.027*
Group-2 versus Group-3	−0.16	−0.53	0.22	0.783
Group-2 versus Group-4	0.23	0.02	0.43	0.031*
Group-2 versus Group-5	0.30	0.13	0.46	0.002*
Group-3 versus Group-4	0.38	0.01	0.76	0.047*
Group-3 versus Group-5	0.45	0.06	0.85	0.028*
Group-4 versus Group-5	0.07	−0.13	0.27	0.864

*Post hoc* Tamhane. The number of rats in each group was 6.

* p<0.05. ADMA=Asymmetric dimethylarginine, CI=Confidence interval

## Discussion

The technique of inducing *H. pylori* infection in rats’ stomachs in this current study was in line with the previous research. The principle in the induction of *H. pylori* infection was to create an environment that supports the growth and colonization of *H. pylori* in the stomach, starting with pre-treatment of streptomycin and omeprazole to inhibit the growth of other bacteria and reduce the level of gastric acidity, respectively [16]. In this study, it can be proven that all rats in *H. pylori* inoculated groups showed positive urease test results.

We noticed some factors attributed to *H. pylori* infection, including high gastric acidity, gastric motility disorder, low albumin level, and biofilm formation [17-20]. High gastric acidity and gastric motility disorder occur due to an increase and disproportion of gastrin and somatostatin activity, respectively [17,18]. Low albumin levels as the result of decreased albumin synthesis, increased albumin degradation, and high transcapillary escape rate in the inflammatory condition [19]. Biofilms are layers of bacterial cells firmly attached to the surface, and can not be easily separated or moved. They are embedded in the self-generated extracellular polymeric substances, including polysaccharides, nucleic acids, and proteins as their protectors. Nutritional sources of *H. pylori* biofilm derived from protein and fat metabolism [20]. Bacterial biofilms in the gastric mucosal epithelium, as *in vivo*, differ structurally and functionally from free-living/planktonic bacteria (*in vitro*). When attached to the gastric mucosal epithelium, bacteria in a biofilm will obtain a regular supply of nutrients from the environment and host so that growth and colonization will be better maintained. The formation of bacterial biofilm leads to resistance to antimicrobial agents [21]. These factors contribute to the performance of agents or drugs in the eradication of *H. pylori*. Hence, the results of *in vitro* studies are often different from the results of *in vivo* studies.

Histological examination is a standard procedure to determine the severity of gastritis objectively based on the USS to assess lymphocyte infiltration, neutrophil activity, glandular atrophy, intestinal metaplasia, and *H. pylori* density. Gastritis is an inflammation of the gastric mucosa in response to gastric injury or infection. Gastritis caused by *H. pylori* is characterized by an increased density of mononuclear cells and polymorphonuclear activation, which generally associated with the density of *H. pylori* bacteria. *H. pylori’s* high density will be directly proportional to the high severity of gastritis [15]. In this study, the severity of gastritis was found in the infected groups.

During the normal protein cycle, ADMA is formed enzymatically from the methylation of arginine remnants through the activity of redox-sensitive S-adenosylmethionine-dependent protein arginine N-methyltransferases and metabolized to citrulline by dimethylarginine dimethylaminohydrolase (DDAH) [22]. ADMA levels reported enhancing during induction of gastric injury with ethanol, indomethacin, cold-stress, and *H. pylori* [23,24]. Those finding is concordant with our study. The production of ADMA in *H. pylori* infection is enhanced by inhibition of antioxidant enzymes activity which leads to the aggregation of reactive oxygen species (ROS) to be cellular oxidative stress and liberation of ­pro-inflammatory cytokines, such as tumor necrosis factor-alpha (TNF-α), interleukin-6 (IL-6), and interferon-gamma [25]. Pro-inflammatory cytokines activate protein arginine N-methyltransferases, an ADMA forming enzyme, and alleviates DDAH activity that degrades ADMA [23]. *H. pylori* infection leads to a life-long inflammation by inducing various gastric mucosal mediators in regulating the mobility of neutrophils, macrophages, and other leukocytes, which associated with gastric mucosal disintegration [26]. The enhancement of ADMA levels is closely related to the gastric mucosal disintegration [27]. Several studies noted that the eradication of *H. pylori* decreases serum ADMA levels [24,28].

We observed that a single administration of *C. striata* extract (Group-3) induced the highest severity of gastritis and led the increment of ADMA levels, as seen in Group-3. These results might be associated with the destruction of *C. striata* due to gastric motility disorder, acid, enzymatically, and biofilm formation. *C. striata* was broken down into albumin, amino acids, fatty acids, and minerals [29,30]. Some albumin clusters, such as thiol and sulfhydryl as an antimicrobial and free radical scavenger, respectively, damaged [31]. Damaged thiol clusters cannot penetrate and diffuse to the biofilm matrix so that the antimicrobial property becomes lost [32]. *H. pylori* requires complex growth media, rich in nutrients, to grow, and form colonies. Once *H. pylori* colonize gastric mucosal hosts, it will utilize host protein and fat metabolism as an energy source. *H. pylori* energy is obtained from amino acids and fatty acids. Amino acids are needed to increase the motility and chemotaxis properties. Motility regulated by chemotaxis is essential in the *H. pylori* growth cycle and colonization [33]. *H. pylori* use aspartate and glutamate to facilitate the production of ammonia by the activity of urease enzyme to reduce gastric acidity level to survive and increase the adhesion to gastric epithelial cells, particularly in the early stage of colonization. Besides, arginine, aspartate, glutamate, and serine also play a role as a source of carbon energy for *H. pylori* in protein synthesis, virulence, and resistance to stress [34]. Biosynthesis of unsaturated fatty acids is needed by *H. pylori* to maintain function and membrane structure. At the same time, minerals are necessary to modify *H. pylori* gene expression so that virulence factors become more effective in forming colonization [35,36]. *C. striata* extract contains major nutrients, such as albumin, amino acids (arginine, lysine, aspartic acid, and glutamic acid), fatty acids, and minerals. Briefly, *in vivo*, a single administration of *C. striata* extract in gastritis *H. pylori* enhances growth and *H. pylori* density because the nutrients of *C. striata* extract might become a source of food or energy for *H. pylori* growth as we observed in Group-3.

The single standard eradication regimen administration in Group-4 showed a decrease in the severity of gastritis and the reduction of ADMA levels compared to Group-3. The standard eradication regimen comprises omeprazole, amoxicillin, and clarithromycin. The existence of omeprazole lowers gastric acidity and makes the antimicrobial performance more optimal [37]. The reduction of gastric acidity level increases gastric amoxicillin concentration and exceeds the minimum inhibitory concentration to *H. pylori* [7,38]. Clarithromycin acts by binding to the 50 s bacterial ribosome subunit to inhibit the synthesis of *H. pylori* protein. A combination of two antimicrobials, amoxicillin and clarithromycin, induce the increase of the eradication rate [7].

Administration, a combination of standard eradication regimen and *C. striata* extract, showed favorable results; this was seen in Group-5, where there was an improvement in the severity of gastritis and decreased ADMA levels. A significant difference was shown in the severity of gastritis, when we compared Group-5 to Group-4 (single administration of standard eradication regimen group). As we noticed in Group-4, soon after omeprazole administration, there was a decrease in gastric acidity, followed by an increase in the performance of both antimicrobials so that there was a decrease of ADMA levels and the improvement of the severity of gastritis. The addition of *C. striata* apparently reduces more ADMA levels, the number of bacteria, and further improves the severity of gastritis, as showed in gastric histological examination. This finding in Group-5 indicates the potentiation effect between standard eradication regimen in eradicating *H. pylori* and *C. striata* extract as an anti-inflammatory, respectively.

Anti-inflammatory properties of *C. striata* extract due to the presence of major nutrients contents such as albumin, amino acids (arginine, lysine, aspartic acid, and glutamic acid), fatty acids, and minerals. Albumin and minerals (zincum, cuprum, and ferrum) possess an antioxidant property that acts as ROS scavenger and cellular protection against oxidative stress [10]. Some amino acids, such as arginine, a substrate in forming NOS, arginase enzymes, and originator of the immune response [39], where lysine, aspartic acid, glutamic acid, and other amino acids perform as antioxidant synergistically with some fatty acids [10]. There are many potent anti-inflammatory properties of these fatty acids. Linoleic acid blocks inflammation by depressing the production of leukotriene B4, a substance that induces the production of TNF-α [40]. Linoleic and arachidonic acids impede the production of pro-inflammatory cytokines such as IL-1β, IL-6, and TNF-α [41]. Oleic and stearic acids act by debilitating the expression of endothelial leukocytes adhesion molecules and reducing the polymorphonuclear leukocytes (PMNs) activity, respectively. The reduction of PMNs activity can prevent ROS release, which can cause tissue damage [42].

## Conclusion

We concluded that a single administration of *C. striata* extract worsens the severity of gastritis and increases serum ADMA levels in the *H. pylori* gastritis rat model. The administration of a combination of standard eradication regimen and *C. striata* extract reduces serum ADMA levels and significantly improves the severity of gastritis compared to the administration of a single *C. striata* extract and a single standard eradication regimen.

## Authors’ Contributions

OKY, AL, SI, and RLK designed and supervised the study. OKY did laboratory works, interpreted the data, drafted, and wrote the manuscript. AL was involved in preparing and critical checking of the manuscript. SI analyzed data and critical checking of the manuscript. RLK participated in the collection of data, laboratory works, and preparation of the manuscript. All authors read and approved the final manuscript.

## Competing Interests

The authors declare that they have no competing interests.

## Publisher’s Note

Veterinary World remains neutral with regard to jurisdictional claims in published institutional affiliation.
